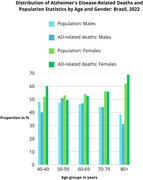# Age and Gender Distribution of Alzheimer's Disease‐Related Deaths: A 2022 Epidemiological Study in Brazil – Who is at greatest risk?

**DOI:** 10.1002/alz70860_107583

**Published:** 2025-12-23

**Authors:** Ana Carolina Sgarioni Viapiana, Vitor Ritt Xavier, Morghana Machado da Rosa, Rodrigo de Cezaro, Cavaler Juvêncio, Gabrielle Guindani Maia, Leonardo Bedatti Koehler, Vitorio Serafim, Ana Júlia Rodrigues Ribeiro, Ana Carolina Gomes Petry, Isabela Alicia Fink

**Affiliations:** ^1^ Fundação Universidade Federal de Ciências da Saúde de Porto Alegre, Porto Alegre, Rio Grande do Sul, Brazil; ^2^ Universidade Federal do Rio Grande do Sul, Porto Alegre, Rio Grande do Sul, Brazil

## Abstract

**Background:**

In 2019, Alzheimer's disease (AD) was the leading cause of dementia‐related deaths in Brazil, accounting for 78.3% of cases, according to the Global Burden of Disease. In this context, understanding the distribution of AD deaths is crucial for developing public health policies. Regarding this, the present study aims to compare Brazilian public health data on AD, analyzing age and gender distribution in both demographic statistics and AD‐related deaths in 2022.

**Method:**

Descriptive study of secondary data from 2022, sourced from the Brazilian Institute of Geography and Statistics (IBGE) for demographics and the Brazilian Mortality Information System (SIM) for AD‐related deaths. Both datasets were stratified by age groups (40–49, 50–59, 60–69, 70–79, and 80 or older) and gender. The female‐to‐male ratio was calculated in both scenarios (population and AD‐related deaths) to evaluate the association between gender and Ad‐related death statistics across age groups.

**Result:**

While the female‐to‐male ratio in the general population, for the 40–49 age group, is 1.07, it rises to 1.5 in AD‐related deaths. In the 50–59, 60–69, and 70–79 age groups, both distributions remain similar. However, among those aged 80 and older, the demographic ratio is 1.6 women per man, increasing to 2.2 in AD‐related deaths, which highlights a real higher risk of Alzheimer's‐related death for women in this age group.

**Conclusion:**

The results support previous findings that women are at a real higher risk of dying from Alzheimer's disease, particularly at ages 80 and older. However, the available data are insufficient to determine whether this increased risk is due to women being more likely to develop Alzheimer's or if they experience more severe forms once diagnosed. Both the frequency and severity may be influenced by factors such as a gender‐based distribution of mental health issues or lower educational attainment, both known risk factors for Alzheimer's. To inform effective public policies, it's essential to improve the public statistics data system. Without more detailed data, it will remain difficult to understand the extent to which gender differences in Alzheimer's mortality are driven by biological, social, or demographic factors. Improved data collection will provide the insights necessary for targeted interventions.